# Gastrocnemius medialis contractile behavior during running differs between simulated Lunar and Martian gravities

**DOI:** 10.1038/s41598-021-00527-9

**Published:** 2021-11-19

**Authors:** Charlotte Richter, Bjoern Braunstein, Benjamin Staeudle, Julia Attias, Alexander Suess, Tobias Weber, Katya N. Mileva, Joern Rittweger, David A. Green, Kirsten Albracht

**Affiliations:** 1 Department of Medical Engineering and Technomathematics, University of Applied Sciences Aachen, Aachen, Germany; 2 Institute of Movement and Neurosciences, German Sport University Cologne, Cologne, Germany; 3 Institute of Biomechanics and Orthopaedics, German Sport University Cologne, Cologne, Germany; 4 Centre for Health and Integrative Physiology in Space (CHIPS), Cologne, Germany; 5 German Research Centre of Elite Sport, Cologne, Germany; 6 Centre of Human and Applied Physiological Sciences, King’s College London, London, UK; 7 European Astronaut Centre (EAC), European Space Agency, Space Medicine Team (HRE-OM), Cologne, Germany; 8 KBR GmbH, Cologne, Germany; 9 School of Applied Sciences, London South Bank University, London, UK; 10 Institute of Aerospace Medicine, German Aerospace Center (DLR), Cologne, Germany; 11 Department of Pediatrics and Adolescent Medicine, University of Cologne, Cologne, Germany; 12 Institute for Bioengineering, University of Applied Sciences Aachen, Aachen, Germany

**Keywords:** Ultrasound, Bone quality and biomechanics, Tendons, Skeletal muscle, Environmental impact

## Abstract

The international partnership of space agencies has agreed to proceed forward to the Moon sustainably. Activities on the Lunar surface (0.16 g) will allow crewmembers to advance the exploration skills needed when expanding human presence to Mars (0.38 g). Whilst data from actual hypogravity activities are limited to the Apollo missions, simulation studies have indicated that ground reaction forces, mechanical work, muscle activation, and joint angles decrease with declining gravity level. However, these alterations in locomotion biomechanics do not necessarily scale to the gravity level, the reduction in gastrocnemius medialis activation even appears to level off around 0.2 g, while muscle activation pattern remains similar. Thus, it is difficult to predict whether gastrocnemius medialis contractile behavior during running on Moon will basically be the same as on Mars. Therefore, this study investigated lower limb joint kinematics and gastrocnemius medialis behavior during running at 1 g, simulated Martian gravity, and simulated Lunar gravity on the vertical treadmill facility. The results indicate that hypogravity-induced alterations in joint kinematics and contractile behavior still persist between simulated running on the Moon and Mars. This contrasts with the concept of a ceiling effect and should be carefully considered when evaluating exercise prescriptions and the transferability of locomotion practiced in Lunar gravity to Martian gravity.

## Introduction

Human space exploration has fascinated mankind since the start of the Space Age in the 1950s. Approximately 50 years after humans first set foot on the Moon, space agencies taking part in the international collaborative Artemis program have agreed to proceed forward to the Moon sustainably. Plans include to building the Lunar Orbital Platform-Gateway, including a Human Lunar Lander, and setting up a permanent surface habitat that may serve as a springboard for future human missions to Mars^1^.

Although the Apollo missions showed that humans can effectively operate in Lunar gravity^2^, with surface stay times of up to 75 h^3^, the data collected during locomotion which would provide useful information about biomechanical alterations required to enable surface activities and the development of evidence-based exercise countermeasures are lacking. Leg muscles, such as the gastrocnemius medialis (GM), that are largely involved in body support and forward acceleration^4^ were observed to be particularly susceptible to atrophy and to architectural changes induced by reduced loading^5,6^. Thus, on Earth as well as on the International Space Station (ISS), running serves as a countermeasure, as the forces that generate both skeletal and muscular loading provide important mechanical stimuli for the musculoskeletal system^7^. However, alterations in gravitational acceleration (g) appear to modify running gait. Thus, ground-based analogues have been developed to study locomotion in simulated hypogravity^8^. However, most hypogravity biomechanical studies have focused on identifying differences with Earth’s gravitational acceleration (1 g)^9,10^.

Previous studies investigating running at 1 g and at simulated hypogravity levels broadly equivalent to Lunar and Martian gravity (0.16‒0.40 g) have indicated reductions in the magnitudes of most gait parameters, such as ground reaction forces^11,12^, mechanical work^13^, estimated joint forces^12^, and muscle activation^12,14^ with decreasing g-level. Similarly, running kinematics, such as ground contact times, cadence^11,12,15^, and lower limb joint angles^15,16^ also tend to reduce with simulated g-level. However, despite the fact that the ankle dorsiflexion angles are smaller when running in simulated hypogravity, the ankle is reported to follow a similar joint movement profile^17^. Furthermore, the lower limb muscle activation patterns^12,14^ and leg stiffness (considered as a linear spring)^11^ are largely preserved.

Moreover, the biomechanical parameters may not necessarily be proportional to the hypogravity level^18^. Indeed, the GM is sensitive to changes in force loading, as evidenced by a reduction in muscle activation, even though it appears that there might be a ceiling effect around 0.2 g^14^. Running at simulated 0.7 g has shown to modulate GM contractile behavior. For instance, at peak series elastic element (SEE) length, where the force acting on the SEE is at its greatest, the GM fascicles operated at longer lengths, with smaller pennation angles but faster shortening velocities^19^. However, whether this pattern occurs in the GM muscle‒tendon unit (MTU) at simulated Martian (0.38 g) and Lunar gravity (0.16 g) is unknown^9^. Thus, whether fascicle‒SEE behavior is sensitive to low hypogravity levels, e.g., when running on the Lunar and Martian surfaces, remains to be determined. Such knowledge is important to assess the transferability of Lunar surface operations to Martian ones.

However, to compare conditions, one must consider the fact that a decrease in the g-level results in the walk-to-run transition occurring at slower absolute speeds but with similar Froude numbers^20–22^. Thus, to achieve running at ‘dynamically similar’ speeds in simulated hypogravity (i.e., at a similar speed relative to the preferred walk-to-run transition speed, PTS) it is suggested to run at the same Froude number and, hence, at a slower speed^22,23^.

Therefore, to determine whether hypogravity-induced modulation of GM fascicle‒SEE interaction is sensitive to running at low hypogravity levels, we have required participants to run at 125% of the PTS at 1 g, in addition to simulated Martian gravity and Lunar gravity, on the vertical treadmill facility (VTF).

Based on the findings of 0.7 g running^19^, it was hypothesized that, at the time of peak SEE length, ankle dorsiflexion and knee flexion are both smaller, whereas GM fascicles are longer, less pennated, and faster in shortening when running in simulated hypogravity vs. 1 g. These alterations in joint kinematics and fascicle‒SEE interaction are expected to persist between simulated Martian and Lunar gravity; although, the question is to what extent and whether the absolute or relative differences in gravity between Moon and Mars surfaces dominate these alterations.

## Results

### Kinetic and spatio-temporal parameters

Participants running at the predefined simulated hypogravity levels of 0.38 g and 0.16 g generated lower mean hypogravity levels, actually corresponding to 32.6 ± 10.3% and 14.8 ± 3.5%, respectively, of the g-levels determined during running at 1 g on a conventional treadmill. Running speeds corresponding to 125% of the participants’ PTS resulted in average running speeds of 2.62 ± 0.08 m s^−1^ at 1 g, 1.80 ± 0.05 m s^−1^ at simulated Martian gravity, and 1.50 ± 0.04 m s^−1^ at simulated Lunar gravity.

**Figure 1:**
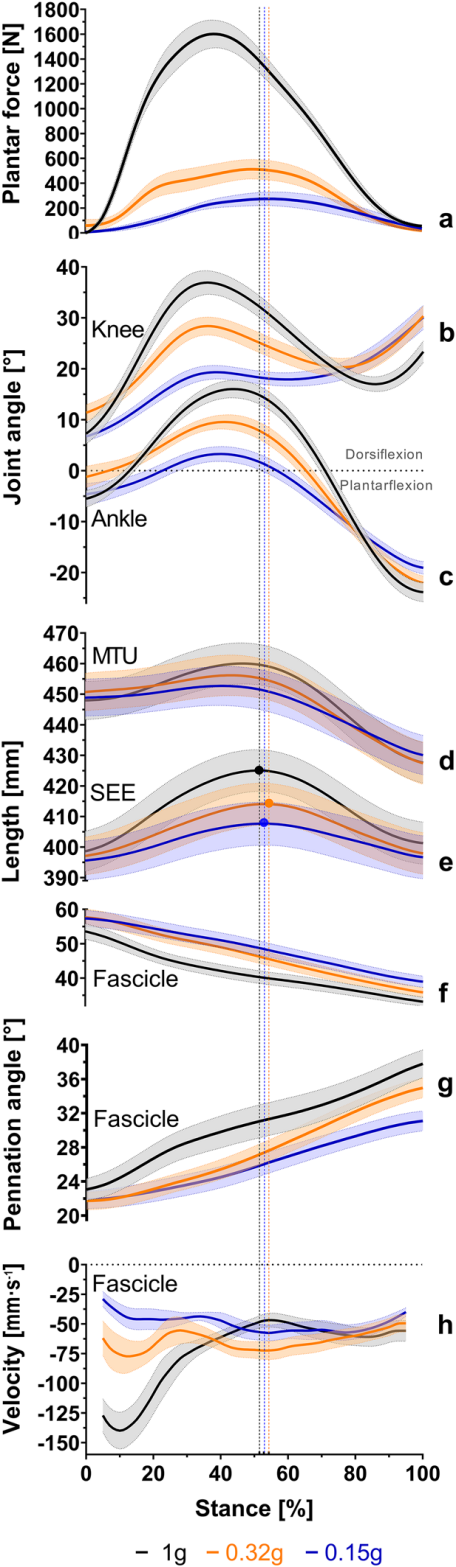
Kinetic, kinematic, gastrocnemius medialis fascicle and series elastic element parameters during the stance phase of running at 1 g, simulated Martian gravity and simulated Lunar gravity. Participants’ average (mean ± standard error) patterns of plantar forces (**a**), knee (**b**) and ankle (**c**) joint angles, and muscle‒tendon unit (**d**) and series elastic element (**e**) lengths as well as muscle fascicle lengths (**f**), pennation angles (**g**), and velocities (**h**) change during the stance phase of running at 1 g (black line), simulated 0.32 g (orange line), and 0.15 g (blue line). The vertical dashed lines mark the point of time at which peak series elastic element length was achieved (in % of stance) at 1 g (black), simulated 0.32 g (orange), and 0.15 g (blue). Please note that the observed hypogravity levels were slightly lower than the actual values for Martian and Lunar gravity. Means and standard errors of the 1 g condition have previously been published by Richter et al.^19^. n = 8 participants.

A significant effect of g-level was noted on peak plantar force, ground contact time, gait cycle duration, cadence, and stride length (Table 1). Peak plantar forces were significantly reduced at both simulated Martian and Lunar gravity compared to 1 g. At simulated Lunar gravity, peak plantar forces were significantly lower than during running at simulated Martian gravity (Table 2, Fig. 1a). Ground-contact times and gait cycle durations were significantly longer at both simulated Martian and Lunar gravity vs. 1 g and were significantly longer at simulated Lunar gravity than at simulated Martian gravity (Table 2). Gait cadence was significantly reduced at both simulated Martian and Lunar gravity compared to 1 g. At simulated Lunar gravity, participants ran at significantly lower cadence than they did at simulated Martian gravity (Table 2). In contrast, despite a significant effect of g-level, no significant post-hoc differences in stride length were observed between 1 g and simulated Martian and Lunar gravity, or between Martian and Lunar gravity (Table 2).

**Table 1:** ANOVA results for kinetic, spatio-temporal, kinematic, gastrocnemius medialis fascicle and series elastic element parameters while participants ran at 125% PTS at 1 g and simulated Martian and Lunar gravity.

Outcomes	1 g		0.32 g		0.15 g		Test statistic	*P*	f(U)
	M	SD	M	SD	M	SD			
Peak plantar force [N]	1612.3	348.3	616.0	159.7	315.7	154.1	F(1.1, 7.8) = 199.6	< 0.0001	5.3
Ground contact time [s]	0.30	0.04	0.38	0.06	0.41	0.08	F(1.3, 8.8) = 39.7	< 0.0001	2.4
Gait cycle duration [s]	0.72	0.05	0.97	0.08	1.18	0.18	F(1.3, 9.3) = 48.3	< 0.0001	2.6
Cadence [steps min^−1^]	83.3	5.9	62.3	4.9	52.0	7.4	F(1.8, 12.8) = 117.8	< 0.0001	4.1
Stride length [m]	1.9 (0.1)		1.7 (0.2)		1.7 (0.3)		χ^2^(2) = 6.8	0.0375	0.7
Ankle joint angle at peak SEE length [°]	15.2	5.1	7.3	4.9	1.5	3.8	F(1.6, 10.9) = 47.5	< 0.0001	2.6
Knee joint angle at peak SEE length [°]	31.9	6.3	24.6	5.5	18.1	3.7	F(1.5, 10.2) = 23.2	0.0003	1.8
Fascicle length at peak SEE length [mm]	40.3	5.8	45.7	5.8	48.5	5.8	F(1.2, 8.2) = 32.7	0.0003	2.2
Pennation angle at peak SEE length [°]	31.2	5.8	27.5	3.6	26.0	3.4	F(1.4, 9.8) = 20.8	0.0006	1.7
Fascicle velocity at peak SEE length [mm s^−1^]	− 49.0 (18.2)		− 72.8 (33.8)		− 52.6 (24.4)		χ^2^(2) = 12.0	0.0011	
Peak SEE length [mm]	425.5	20.8	414.3	20.5	407.8	21.3	F(1.4, 9.8) = 47.0	< 0.0001	2.6
Time of peak SEE length [% Stance]	52.0 (11.8)		53.5 (7.8)		54.5 (5.3)		χ^2^(2) = 0.8	0.7147	
MTU length at peak SEE length [mm]	460.1	20.5	454.9	20.2	451.4	20.0	F(1.6, 11.2) = 32.7	< 0.0001	2.2
MTU elongation [mm]	13.0	2.8	7.0	3.3	5.3	2.6	F(1.5, 10.2) = 39.6	< .0.0001	2.4
Fascicle shortening (during SEE elongation) [mm]	13.3	3.3	12.0	2.9	8.9	3.4	F(2.0, 13.9) = 17.5	0.0002	1.6
Delta pennation angle (during SEE elongation) [°]	8.1	3.2	5.8	1.6	4.2	1.4	F(1.4, 9.7) = 16.7	0.0014	1.5
Average fascicle velocity (during SEE elongation) [mm s^-1^]	− 97.0	20.8	− 64.6	13.5	− 44.7	12.0	F(1.7, 12.0) = 75.9	< 0.0001	3.3

*PTS* preferred walk-to-run transition speed, *M* mean, *SD* standard deviation, *P* result of the ANOVA (F-statistic) or Friedman test (χ^2^) indicating a significant effect of g-level (α set to 0.05), *f(U)* effect size ANOVA; Results of the Friedman test are presented as medians (interquartile ranges). Peak SEE length at 1 g, simulated 0.32 g (Mars), and 0.15 g (Moon) occurred at 52% ± 8%, 54% ± 4% and 53% ± 5% of stance, respectively. Mean and standard deviation for ground contact time, cadence, and joint angles for the 1 g condition have been previously published by Richter et al. ^19^. n = 8.

**Table 2:** Post-hoc results for kinetic, spatio-temporal, kinematic, gastrocnemius medialis fascicle and series elastic element parameters while participants ran at 125% PTS at 1 g and simulated Martian and Lunar gravity.

Outcomes	1 g vs. 0.32 g					1 g vs. 0.15 g					0.32 g vs. 0.15 g				
	M	SD	95% CI	*P*	d	M	SD	95% CI	*P*	d	M	SD	95% CI	*P*	d
Peak plantar force [N]	− 996.4	221.9	− 1227.4; − 765.3	< 0.0001	− 3.7	− 1296.6	239.5	− 1546.0; − 1047.3	< 0.0001	− 4.8	− 300.3	64.8	− 367.8; − 232.8	< 0.0001	− 1.9
Ground contact time [s]	0.08	0.03	0.05; 0.11	0.0006	1.7	0.11	0.05	0.06; 0.16	0.0008	1.9	0.03	0.03	0.01; 0.06	0.0168	0.5
Gait cycle duration [s]	0.25	0.07	0.17; 0.32	< 0.0001	3.9	0.45	0.16	0.29; 0.62	0.0002	3.4	0.21	0.14	0.06; 0.36	0.0116	1.5
Cadence [steps min^−1^]	− 21.0	5.6	− 26.9; − 15.1	< 0.0001	− 3.9	− 31.2	6.7	− 38.2; − 24.3	< 0.0001	− 4.7	− 10.3	5.2	− 15.7; − 4.9	0.0020	− 1.6
Stride length [m]	− 0.2 (0.2)			0.0733	− 1.4	− 0.1 (0.3)			0.0733	− 0.8	− 0.01 (0.2)			> 0.9999	0.1
Ankle joint angle at peak SEE length [°]	− 7.9	3.3	− 11.3; − 4.5	0.0006	− 1.6	− 13.7	4.9	− 18.9; − 8.6	0.0003	− 3.0	− 5.8	3.6	− 9.5; − 2.1	0.0063	− 1.3
Knee joint angle at peak SEE length [°]	− 7.3	4.9	− 12.4; − 2.2	0.0096	− 1.2	− 13.9	7.3	− 21.4; − 6.3	0.0026	− 2.7	− 6.5	4.7	− 11.4; − 1.6	0.0138	− 1.4
Fascicle length at peak SEE length [mm]	5.4	1.5	3.9; 7.0	< 0.0001	0.9	8.1	3.8	4.2; 12.1	0.0013	1.4	2.7	2.9	− 0.3; 5.8	0.0758	0.5
Pennation angle at peak SEE length [°]	− 3.7	2.3	− 6.1; − 1.2	0.0073	− 0.8	− 5.2	2.9	− 8.2; − 2.1	0.0039	− 1.1	− 1.5	1.5	− 3.1; 0.1	0.0630	− 0.4
Fascicle velocity at peak SEE length [mm s− ^1^]	− 25.2 (25.6)			0.0081	1.4	− 3.3 (17.6)			>0 .9999	0.3	13.8 (24.9)			0.0081	− 0.9
Peak SEE length [mm]	− 11.2	3.8	− 15.1; − 7.3	0.0002	− 0.5	− 17.7	6.7	− 24.6; − 10.7	0.0003	− 0.8	− 6.5	4.8	− 11.5; − 1.4	0.0165	− 0.3
Time of peak SEE length [% Stance]	− 0.5 (11.3)				0.5	2.5 (7.0)				0.2	1.0 (7.0)				− 0.3
MTU length at peak SEE length [mm]	− 5.2	2.5	− 7.8; − 2.6	0.0016	− 0.3	− 8.6	3.7	− 12.5; − 4.8	0.0008	− 0.4	− 3.5	2.8	− 6.4; − 0.6	0.0226	− 0.2
MTU elongation [mm]	− 6.0	2.3	− 8.3; − 3.6	0.0004	− 1.9	− 7.7	3.2	− 11.0; − 4.3	0.0007	− 2.8	− 1.7	2.0	− 3.8; 0.4	0.1051	− 0.6
Fascicle shortening (during SEE elongation) [mm]	− 1.3	2.1	− 3.5; 0.8	0.2305	− 0.4	− 4.4	2.2	− 6.7; − 2.1	0.0022	− 1.3	− 3.1	2.2	− 5.3; − 0.8	0.0124	− 1.0
Delta pennation angle (during SEE elongation) [°]	− 2.3	2.0	− 4.5; − 0.2	0.0342	− 0.9	− 4.0	2.4	− 6.5; − 1.5	0.0057	− 1.6	− 1.6	1.2	− 2.9; − 0.4	0.0156	− 1.1
Average fascicle velocity (during SEE elongation) [mm s− ^1^]	32.3	13.8	18.0; 46.7	0.0008	− 1.8	52.3	12.7	39.1; 65.5	< 0.0001	− 3.1	20.0	9.4	10.2; 29.8	0.0014	− 1.6

*PTS* preferred walk-to-run transition speed; *M* mean; *SD* standard deviation; *CI* Confidence Interval; *P* result of the post-hoc test indicating a significant effect between conditions (α set to 0.05); *d* effect size (Cohen’s d) for the post-hoc test. Results of the Friedman test are presented as medians (interquartile ranges). Peak SEE length at 1 g and simulated 0.32 g (Mars), and 0.15 g (Moon) occurred at 52% ± 8%, 54% ± 4% and 53% ± 5% of stance, respectively. n = 8.

### Joint kinematics

The participants’ average knee (Fig. 1b) and ankle (Fig. 1c) joint movement profiles (plotted as a function of stance phase) were suppressed when running occurred at both simulated Lunar gravity and Martian gravity vs. 1 g.

**Figure 2:**
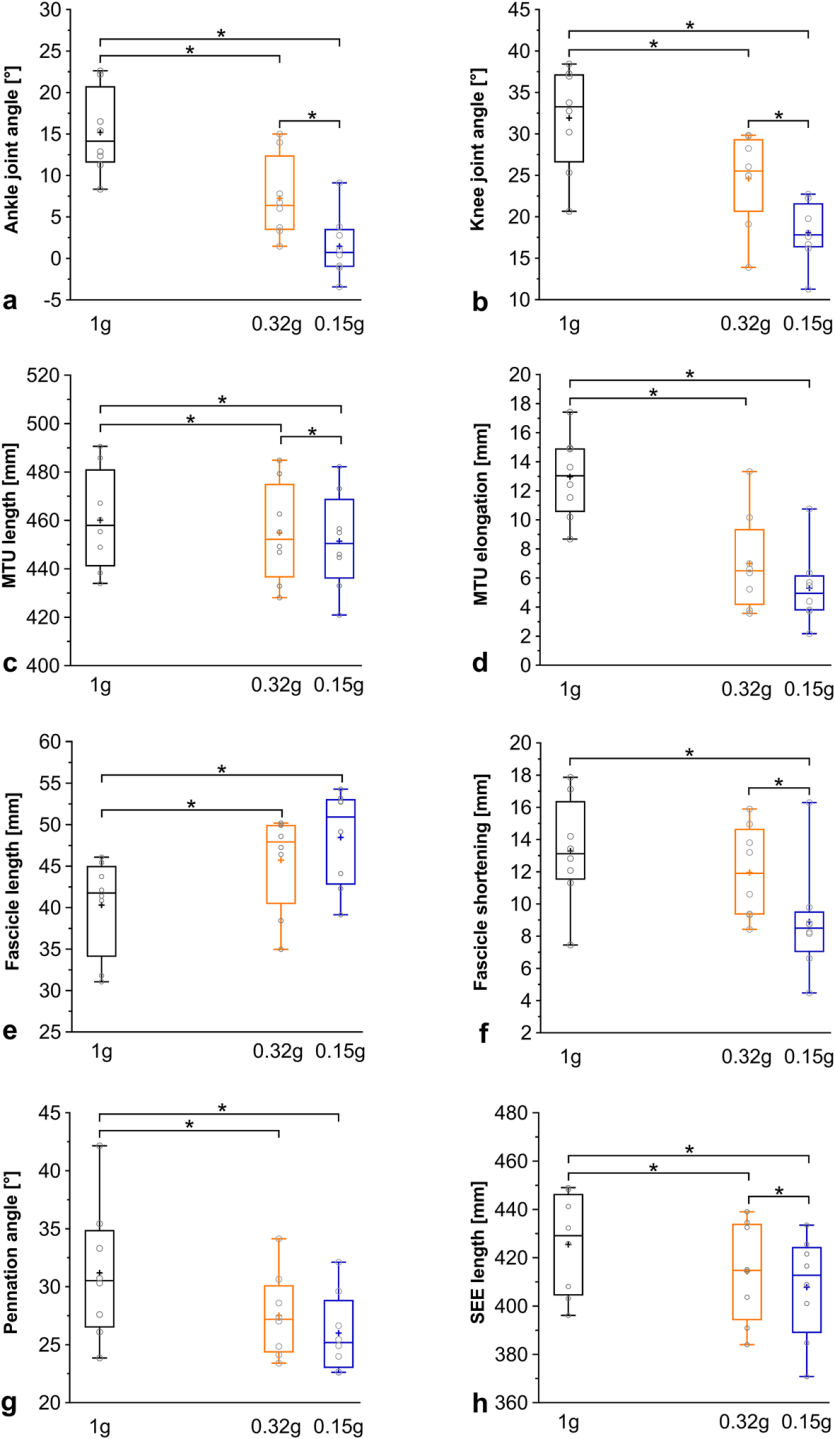
Gastrocnemius medialis fascicle and series elastic element behavior at the time of peak series elastic element length when running at 1 g, simulated Martian gravity, and simulated Lunar gravity. Ankle joint angle (**a**), knee joint angle (**b**), muscle‒tendon unit length (**c**), fascicle length (**e**), pennation angle (**g**) and series elastic element length (**h**) at the time of the peak series elastic element length as well as muscle‒tendon unit elongation (**d**) and fascicle shortening during series elastic element elongation (**f**) when running at 1 g (black box), 0.32 g (orange box) and 0.15 g (blue box). Please note that the observed hypogravity levels were slightly lower than the actual values for Martian and Lunar gravity. The lower and upper parts of the box represent the first and third quartile, respectively. The length of the whisker represents the minimum and maximum values. The horizontal line in the box represents the statistical median of the sample; + the mean of the sample; ○ individual data points; *significantly different (Tukey post-hoc, *p* ≤ 0.05). The boxplots of the 1 g condition in (**c**,**e**,**g**,**h**) have previously been published by Richter et al.^19^. n = 8 participants.

There was a significant effect of g-level on ankle joint angle and knee joint angle when the peak SEE length was reached (Table 1). Ankle dorsiflexion (Fig. 2a) and knee flexion (Fig. 2b) angles at peak SEE length were both significantly smaller during running at simulated Martian and Lunar gravity compared to 1 g. At simulated Lunar gravity, the ankle joint was also significantly less dorsiflexed, and the knee joint was significantly less flexed than at simulated Martian gravity (Table 2).

### GM muscle and SEE parameters

GM muscle–SEE parameters, such as MTU length (Fig. 1d), SEE length (Fig. 1e), fascicle length (Fig. 1f), pennation angle (Fig. 1g) and fascicle velocity (Fig. 1h) (plotted as a function of stance phase), were modulated when running was performed at 1 g vs. simulated Martian gravity and Lunar gravity.

A significant effect of g-level was observed on GM fascicle length, pennation angle, and fascicle velocity at peak SEE length (Table 1). At the time of peak SEE length, the fascicles operated at a significantly longer length (Fig. 2e) but a smaller pennation angle (Fig. 2g) at both simulated Martian and Lunar gravity compared to 1 g. However, no significant differences were noted between simulated Martian gravity and Lunar gravity (Table 2). In contrast, while the fascicles shortened significantly faster (at the time of peak SEE length) at simulated Martian gravity compared to 1 g, no significant differences were observed at simulated Lunar gravity vs. 1 g. The fascicle velocity was significantly slower when running was performed at simulated Lunar vs. Martian gravity (Table 2).

Furthermore, there was a significant effect of g-level on SEE length and MTU length at the time of peak SEE length, as well as on MTU elongation (Table 1). The time point at which peak SEE length was reached (51.5 ± 7.5%, 54.3 ± 4.0%, and 52.8 ± 5.2% of stance at 1 g, Martian gravity, and Lunar gravity, respectively) did not differ between g-levels (Table 1). Both the peak SEE length (Fig. 2h) and MTU length at the time of peak SEE length (Fig. 2c) were significantly shorter when running was conducted at simulated Martian and Lunar gravity compared to 1 g and when running was performed at simulated Lunar gravity compared to simulated Martian gravity (Table 2). MTU elongation (Fig. 2d) was significantly lower in both simulated Martian and Lunar gravity vs. 1 g. However, no differences were observed between the findings for simulated Mars and Moon (Table 2).

The g-level also had a significant effect on fascicle shortening, the delta in pennation angle, and average fascicle velocity during SEE elongation (from touch down to peak SEE length) (Table 1). Fascicle shortening (Fig. 2f) showed no significant differences for running at 1 g and simulated Martian gravity, but showed significant reductions when running at simulated Lunar gravity compared to 1 g and simulated Martian gravity (Table 2). Delta pennation angle and average fascicle velocity between touchdown and peak SEE length were both significantly reduced at simulated Martian and Lunar gravity vs. 1 g (Table 2). Running at simulated Lunar gravity significantly reduced delta pennation angle and average fascicle velocity compared to simulated Martian gravity (Table 2).

## Discussion

The main findings were that spatio-temporal, joint kinematic and most muscle‒SEE outcomes during running at 125% PTS are affected by g-level. Decreasing g-level from 1 g to simulated Martian and Lunar gravity resulted in prolonged ground contact times, decreased cadence, smaller ankle dorsiflexion and knee flexion angles at the time of peak SEE length, shorter peak SEE length, and lower delta in pennation angle and average fascicle velocity during SEE elongation. Fascicle shortening during SEE elongation did not differ between 1 g vs. Martian gravity but was significantly reduced in Lunar gravity vs. Martian gravity and 1 g. These outcomes appear to be sensitive to low hypogravity levels and, thus, indicate that there may be a Martian vs. Lunar effect. In addition, albeit not statistically significant, at peak SEE length, fascicles operated at longer lengths and smaller pennation angles in simulated Lunar gravity as compared toMartian gravity.

The plantar force data acquired in the present study suggest that the participants actually ran at slightly lower hypogravity levels than originally intended in the experimental set-up (0.32 g vs. 0.38 g and 0.15 g vs. 0.16 g). According to the systematic review by Richter et al.^9^ the observed hypogravity levels are still in the range that has been defined for simulated Martian gravity (0.3‒0.4 g) and Lunar gravity (0.1‒0.2 g). Therefore, and in light of the fact that this is a pilot study, we do not expect this deviation from the actual values for Lunar and Martian gravities to strongly affect the overall interpretation of our results.

Running in simulated Martian and Lunar gravity resulted in prolonged ground contact times and decreased cadence at constant stride length, whereas previous studies investigating running at approximately 3.00 m s^−1^ at simulated hypogravity reveal shorter ground contact times^11,12,15,24,25^ and increased stride lengths^24,25^ compared to 1 g. This contradicts the present results. However, it should be noted that, in the present study, participants ran at almost half of these speeds (1.8 m s^−1^ and 1.5 m s^−1^ at simulated Martian and Lunar gravity, respectively), because running speeds were intentionally decreased with decreasing g-level by adjusting running speeds to the same Froude number. This was done to ensure that subjects run at similar speeds relative to the PTS, which are considered to be mechanically equivalent independent of the gravity level. Moreover, running at the same Froude number usually produces equal relative stride length^26^. Thus, maintenance of stride length could be attributed to the present methodological approach of running at a mechanically equivalent speed at each g-level.

However, ankle and knee joint kinematics were modulated by hypogravity running, demonstrating modifications in the participants’ running pattern in relation to 1 g. We did indeed expect ankle dorsiflexion and knee flexion at peak SEE length to become smaller with lower simulated hypogravity levels, as similar findings have been reported in previous hypogravity studies^15,17,24^. However, we did not expect that the small absolute difference in the hypogravity level between simulated Martian and Lunar gravity would produce reductions in ankle dorsiflexion and knee flexion angles, which are almost as large as the reductions in these joint angles between 1 g and Martian gravity. Nevertheless, when looking at the relative difference between the two hypogravity levels, the distinct changes in joint kinematic characteristics between simulated running on Mars and Moon are less surprising, given that Martian gravity is more than twice as much as Lunar gravity.

In the present study, participants’ knee joint was less flexed the lower the hypogravity level, which supports the idea that participants adapt their running pattern according to the much lower energy absorption required with decreasing hypogravity levels^15^. In addition, the significantly smaller knee flexion angles at peak SEE length could also be the result of the reduced external work required to lift and forward-accelerate the body’s center of mass when running in simulated hypogravity^13^. This effect could be even more pronounced by the fact that the present participants were not vertical but, instead, were horizontally suspended on the VTF. Thus, participants presumably counteracted their less flexed knee joints (which is likely caused by both reduced g-levels and unusual body positions) by placing their ankle joints in a position involving a smaller dorsiflexion. In fact, in the present study, despite a similar ankle joint angle at initial contact when running at simulated Lunar gravity vs. 1 g, in the subsequent stages of the stance phase, ankle dorsiflexion angles were found to be much smaller. This is also in alignment with previous hypogravity studies^15,24^, which suggest that participants shift to a forefoot striking pattern^15^.

Thus, from a joint kinematic point of view, running at simulated Lunar and Martian gravity is not equivalent to running at 1 g; further, running at simulated Lunar gravity differs from running at simulated Martian gravity, which, in turn, does not concur with the idea of a ceiling effect. This is further supported by the large effect sizes that were identified for lower limb joint angles.

As MTU lengths were calculated on the basis of ankle and knee joint angles, it is unsurprising that significant g-level effects were also observed for MTU lengths determined at the time of peak SEE length. The fact that MTU lengths become shorter during running at simulated hypogravity suggests that smaller ankle dorsiflexion compensates for the less-flexed knee joint, as was already observed when running in simulated 0.7 g^19^. In addition, lower external forces acting on the SEE during hypogravity running presumably generate shorter peak lengths and, thus, confirm anticipated results that peak SEE length significantly decreases with hypogravity level. Shorter peak SEE lengths, as a function of g-level, indicate a reduced storage of elastic strain energy^27^. Thus, the smaller elastic stretch may also be a functional adaptation to the lower mechanical energy storage requirements of running on the simulated surfaces of the Moon as compared to those of Mars^13^.

Gastrocnemius medialis contractile behavior during running in simulated hypogravity appears to be more variable than joint kinematics or SEE length modulation. However, as expected, the present study showed that fascicles operated at longer lengths and smaller pennation angles in simulated Martian and Lunar gravity compared to 1 g, which is similar to running in simulated 0.7 g using the VTF^19^. Corresponding effect sizes for the comparisons to 1 g were large.

Yet, contrary to the present hypothesis that significant alterations persist between Mars and Moon, fascicle length and pennation angle at the time of peak SEE length did not differ significantly for the simulated Martian and Lunar running. This, in turn, suggests that for fascicle’s operating length, there might exist a ceiling effect that is similar to the one originally introduced by Mercer et al.^14^ for the reduction in muscle activation, which was stabilized around 0.2 g. Albeit not statistically significant, at the time of peak SEE length, fascicles operated at 3  ± 3 mm longer lengths and 2° ± 2° smaller pennation angles in simulated Lunar gravity than in Martian gravity, still representing effect sizes of d = 0.5 and − 0.4, respectively. Thus, further research is warranted using ultrasonography combined with measures of muscle activation and ideally including a larger sample size.

With regard to fascicle behavior, it should also be highlighted that, during the SEE elongation (where muscular forces are naturally required to stretch the SEE and, thus, to store elastic energy), fascicle shortening, average shortening velocity, and the delta in pennation angle were significantly reduced in hypogravity as compared to 1 g; more importantly, they were also reduced for simulated Lunar in relation to Martian gravity, as additionally indicated by the overall large effect sizes. Such alterations in GM contractile behavior, in turn, point to functional adaptations associated with hypogravity running.

For instance, a lower average shortening velocity, which may be associated with the longer ground contact times, suggests an enhanced force generation ability of the GM^28^. In 1 g, GM contractile behavior adapts when switching from a walking to a running gait^29^. However, no change in fascicle velocity is observed when running speeds are further increased^29,30^. The observation that the GM works on a similar part of the force–velocity relationship across various steady-state running speeds^29,30^, however, appears to not account for conditions of simulated hypogravity when running speeds are intentionally decreased to match the Froude number. Thus, to determine whether the observed decrease in fascicle velocity can be solely attributed to the decrease in g-level or in running speed requires further studies.

As discussed above, the shorter peak SEE lengths observed during running in hypogravity might be a part of the functional adaptations to the lower mechanical work output^13^ (the muscle’s work or energy output is roughly proportional to cumulative SEE force multiplied by the change in muscle length). However, this is not the only adaptation that might influence the mechanical work output of the muscle. Reduced GM fascicle shortening along with reduced delta in GM pennation angle is observed during the SEE elongation phase when reducing from simulated Martian to Lunar gravity. This means that the muscle shortening (the combined effect of fascicle length and pennation angle) also tends to be reduced at lower g-levels, which might be another way for the muscle to reduce its overall mechanical work output (by reducing not only the force, as described above, but also its change in length during every stance phase). Interestingly, when reducing simulated g-levels from Earth to Mars to Moon, peak SEE length (and, thus, implied SEE force) appears to reduce first, while fascicle shortening mainly reduces at lower g-levels (e.g. between Martian and Lunar gravity). This might be interpreted such that, when reducing load, the muscle tends to reduce its mechanical work output first via reducing forces (and with it elastic energy stored in the SEE) before reducing the extent to which it is shortened.

In fact, it appears that running in simulated hypogravity in-part impairs the MTU’s stretch–shortening cycle. Plyometric-type exercises appear to be very effective for maintaining the stretch shortening cycle efficacy^31,32^ as they induce relatively high vertical ground reaction forces and thus higher magnitudes of tissue strain^33^. For instance, peak vertical ground reaction forces have been revealed to be negatively related to simulated hypogravity level, but positively to hopping height. Moreover, submaximal hopping (> 15 cm height of flight) in simulated Lunar and Martian gravity is associated with forces that are similar to standing and running on Earth, respectively^32^. This may be why skipping and plyometric training, have been suggested as the preferred gait on the Moon^13^ and a promising countermeasure for preventing musculoskeletal deconditioning^32,33^, respectively. One innovative gravity-independent countermeasure is spring-loaded horizontal jumping, but its applicability in space remains to be evaluated^31^.

In addition, it can be argued that achieving a terrestrial-like fascicle‒SEE behavior, and, thus, having similar stimuli exerted on the GM muscle, is also a valid goal for effective running countermeasure exercises. To achieve this, the lower the hypogravity level, the more external loading that needs to be applied as compensation. In full microgravity, like on ISS, crewmembers strap themselves to a treadmill via a harness-based subject loading system^34^. To achieve terrestrial loading in such a setting, the crewmembers’ full equivalent body weight force would have to be applied on their harness. However, due to harness discomfort, crewmembers typically limit their applied external loading to about 70% equivalent body weight^35^ even if the bungee system would allow applying higher loads.

On Mars, crewmembers will be exposed to a force of 0.38 g, which corresponds to 38% equivalent body weight. Therefore, a harness loading of around 60–70% bodyweight, which is similarly tolerable as the typical loading used on ISS^35^, should be able to effectively compensate for reduced gravity level and result in an external loading that is in the range of full body weight on Earth. In Lunar gravity, the force of 0.16 g acting on the crewmembers’ body will most likely be insufficient to reach their full body weight at a similar harness loading, only adding up to 75–85% body weight. For a Lunar habitat scenario, if this resulting loading is regarded as too low, one might consider complementing the harness-based subject loading system by wearing an additional weight vest. However, to add the missing 15% equivalent body weight loading in Lunar gravity, the weight vest would have to be in the mass range of the crewmember’s personal body mass, which will likely create considerable discomfort through its inertial behavior in response to the crewmember’s running motion. Nevertheless, determination of the optimal body weight loading in hypogravity conditions should be examined in future research. Additionally, studies should also investigate whether crewmembers exposed to 0.16 g could carry equipment that is approximately six times as heavy as on Earth without any risks after their GM behavior has functionally adapted in response to the lower musculoskeletal loading.

In conclusion, simulated hypogravity running (Martian and Lunar gravity) as compared to running at 1 g induced alterations in joint kinematics (e.g., smaller ankle dorsiflexion and knee flexion angles at peak SEE length) and GM contractile behavior (e.g. longer fascicles and smaller pennation angles at peak SEE length and slower average shortening velocities during SEE elongation). Moreover, joint kinematics and GM contractile behavior during running in simulated Lunar gravity are not equivalent to those for Mars, as indicated by their sensitivity to the small absolute difference but, more importantly, large relative difference in gravity between Moon and Mars surfaces. This could impair the transferability of Lunar to Martian surface operations that involve locomotion. Finally, while crewmembers performing running countermeasures on Mars would be able to apply full body weight loading at a similar perceived harness discomfort as that on ISS, crewmembers exposed to Lunar gravity would have to apply greater external loading to induce mechanical stimuli that are similar to those experienced on Earth.

## Methods

The methods used in the present study are the same as reported in a previous publication^19^, except for the hypogravity levels, some additional outcome parameters, and the statistical analysis. Some parts that are identical to the methods in Richter et al.^19^ have been shortened.

### Participants

Eight healthy male volunteers (31.9s ± 4.7 years, 178.4  ± 5.7 cm height, 94  ± 6 cm leg lengths, and 73.5  ± 7.3 kg body masses) were examined medically, and informed written consent to participate in this study was obtained from them. This study received approval from the ‘Ärztekammer Nordrhein’ Ethical Committee of Düsseldorf, Germany, in accordance with the ethical standards of the 1964 Helsinki declaration. Exclusion criteria included the occurrence of any cardiovascular, musculoskeletal, or neurological disorders within two years of the study.

### Study design and experimental protocol

**Figure 3:**
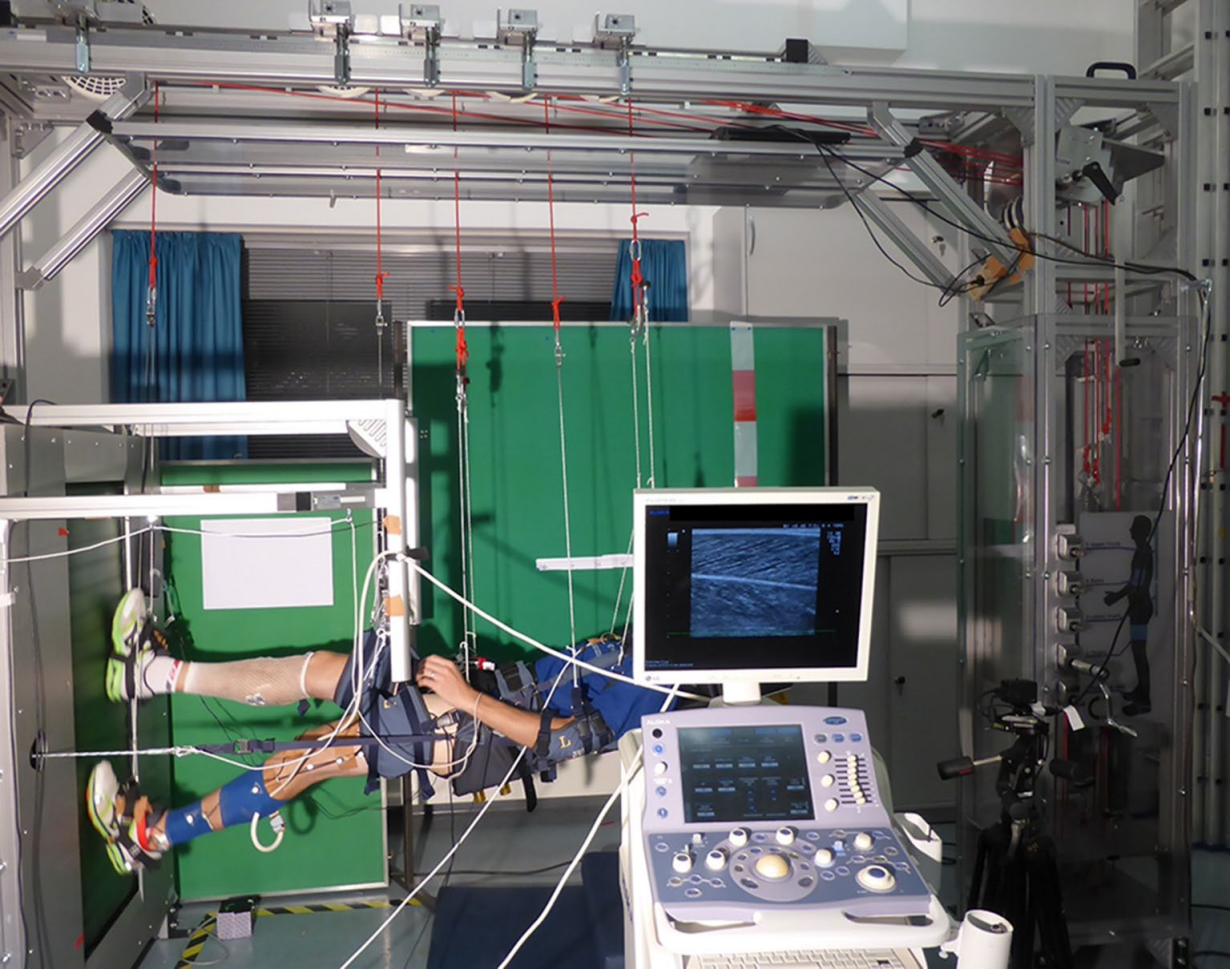
VTF Experimental set-up. Participant being suspended horizontally on the vertical treadmill facility (VTF) with an ultrasound transducer attached to the midbelly of the GM muscle and electrogoniometers placed over the knee and ankle joint to record the respective joint angles. Photo credit: Charlotte Richter; informed consent was obtained to publish this photograph.

The participants visited the laboratory on a single occasion and ran on the vertical treadmill facility (VTF; Arsalis, Glabais, Belgium, Fig. 3) at simulated Martian and Lunar gravity (randomized order), in addition to running on a conventional treadmill at 1 g. Before each running trial, the participants familiarized themselves (~ 4 min) until they had acclimatized to the simulated gravity level and the predefined running speed. After another two minutes of accommodation time^36^, data were collected for 30 s. As this protocol was conducted as part of a larger study, the corresponding data of all eight participants for 1 g have already been included as a control condition in a recent publication^19^.

To obtain mechanically equivalent running speeds at all tested g-levels, running speeds were defined as 125% of the preferred walk-to-run transition speed (PTS). This was estimated by fitting an exponential regression equation $({PTS}_{FR} (a)=1.183{e}^{-5.952a}+0.4745)$ with a least-squares method (r^2^ = 0.99) to the data provided by Kram et al.^20^ using the resulting acceleration (*a*) as the independent variable. By accounting for each participant’s leg length (l), the individual $PTS(a)=\sqrt{{PTS}_{FR}\left(a\right) \cdot a\cdot l}$ was determined. A running gait was ensured by adding 25% to this PTS, and this resulted in participants running at predefined speeds of 2.62 ± 0.08 m s^−1^ at 1 g, 1.80 ± 0.05 m s^−1^ at simulated Martian gravity, and 1.50 ± 0.04 m s^−1^ at simulated Lunar gravity.

### Data collection

To determine the stance phase (touchdown to toe-off), each participants’ plantar force was acquired at 83 Hz via shoe insoles (novel GmbH, loadsol^®^ version 1.4.60, Munich, Germany). The gait cycle events were automatically detected via a custom-made script (MATLAB R2018a, MathWorks, Inc., Natick, United States) that used a 20 N force threshold for 0.1 s.

Knee and ankle joint angle data were sampled at 1500 Hz via the TeleMyo 2400 G2 Telemetry System (Noraxon USA., Inc., Scottsdale, USA) and MyoResearch XP software (Master Edition 1.08.16) using a twin-axis electrogoniometer (Penny and Giles Biometrics Ltd., Blackwood Gwent, UK) for the knee and a custom-made 2D-electrogoniometer for the ankle joint. Electrogoniometer and loadsol signals were time-synchronized by recording a rectangular TTL pulse generated by pressing on a custom-made pedal. Before each running trial, the electrogoniometers were zeroed when the participant was in an anatomical neutral position (standing).

**Figure 4:**
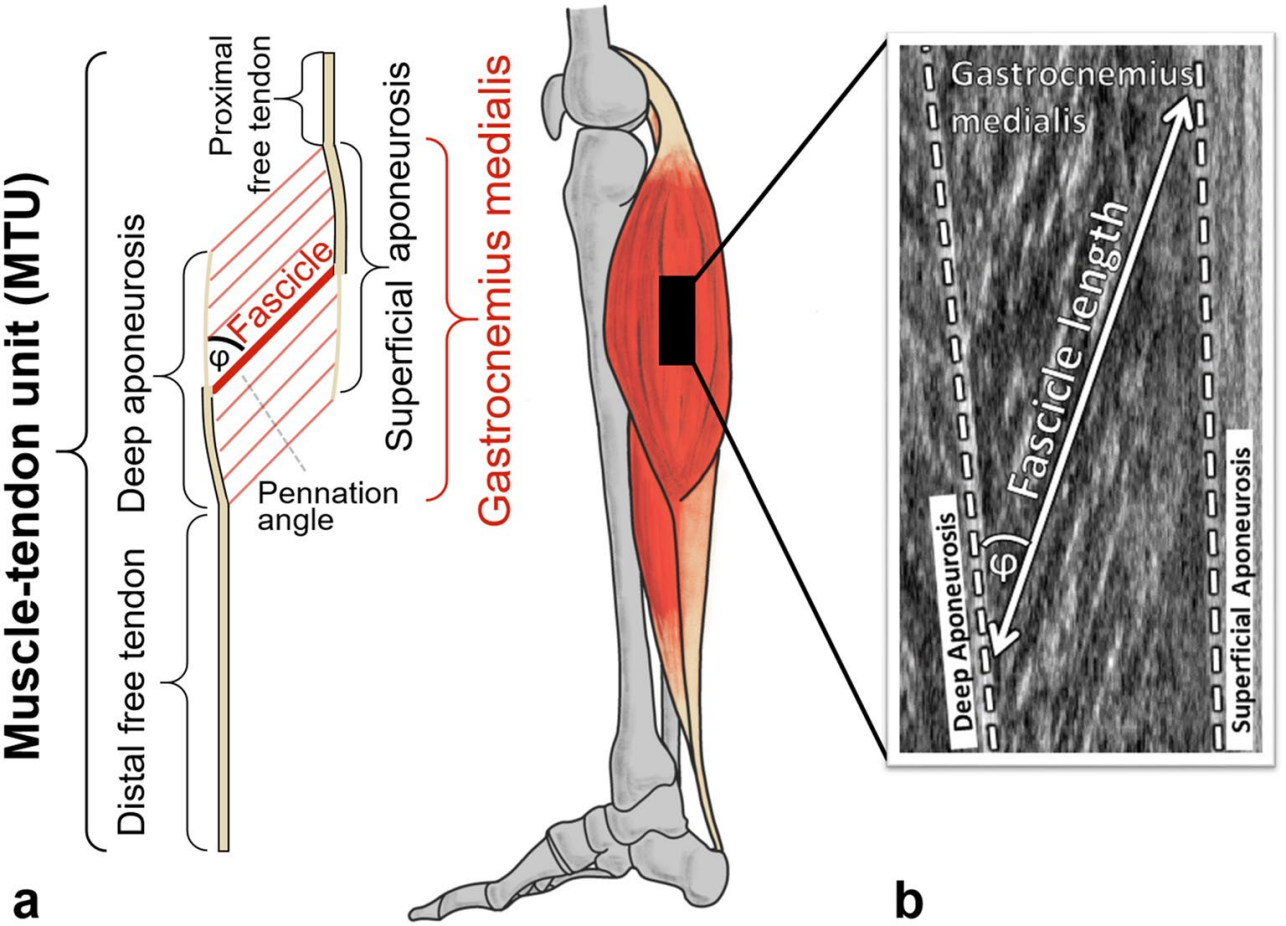
Schematic and anatomical muscle–tendon unit model (**a**) in addition to an actual annotated ultrasound image of the gastrocnemius medialis (**b**). The series elastic element consists of all tendon-like elements, i.e. free tendon and aponeuroses, as shown in beige (**a**). The pennation angle (φ) of the muscle fascicles is defined with respect to the deep aponeurosis. Fascicle length is measured as the length following the pennation between the deep and the superficial aponeuroses (**b**).

B-mode ultrasonography (Prosound α7, ALOKA, Tokyo, Japan) was used to image the GM fascicles at a frame rate of 73 Hz. The T-shaped 6-cm linear array transducer (13 MHz) was positioned inside a custom-made cast over the GM mid-belly and secured with elastic Velcro. The ultrasound recordings and electrogoniometer signals were time-synchronized via a rectangular TTL pulse generated by a hand switch, which was recorded on the electrocardiography channel of the ultrasound device, and the MyoResearch XP software. GM fascicle lengths (distance between the insertions into the superficial and the deep aponeuroses) and pennation angles (angle between the fascicle and the deep aponeurosis) were quantified (Fig. 4) and, where appropriate, were manually corrected using a semi-automatic tracking algorithm (UltraTrack Software, version 4.2)^37^.

The SEE length (Achilles tendon, aponeuroses and proximal tendon; Fig. 4), was calculated by multiplying the muscle fascicle lengths by the cosine of its pennation angle and then subtracting that value from the MTU length^38^. Muscle−tendon unit length was calculated by a multiple linear regression equation^39^ using the participant’s shank length and their knee and ankle joint angles.

### Data processing

For each participant and each outcome measured at each g-level, eight consecutive left foot stance phases were analyzed via a custom-made script (MATLAB R2018a, MathWorks, Inc., Natick, United States). Prior to being resampled to 101 data points per stance phase, ultrasound data were smoothed with a five-point moving average, whereas electrogoniometer signals were smoothed with a fifth-order Butterworth low-pass filter at a 10-Hz cut-off frequency. Fascicle velocities were calculated as the time derivative of the respective length using the central difference method^40^.

To estimate the loading achieved on the VTF, average simulated g-levels over the stance phase were calculated via plantar force and impulse, and expressed as a percentage of the average g-levels that were determined similarly during running on a conventional treadmill. Peak plantar force was defined as the maximum force value observed during stance. Ground-contact times and gait cycle durations were calculated as the time between left foot touchdown and toe-off and between left foot touchdown to the next ipsilateral touchdown, respectively. Cadence was defined as steps (gait cycle duration) per minute. Stride lengths were determined by multiplying gait cycle durations with running velocities. Ankle and knee joint angles as well as SEE-, fascicle-, and MTU lengths in addition to fascicle pennation angles and velocities were determined at the time of the peak SEE length, when the force acting on the SEE is at its greatest. MTU elongation was calculated as the difference between touchdown and peak length. Fascicle shortening and changes in pennation angle that occurred during SEE elongation were calculated by subtracting the respective values at touchdown from the values measured at peak SEE length. Average fascicle velocity was determined for the phase of SEE elongation.

### Statistical analysis

Data distribution for all outcome measures was assessed using the Shapiro–Wilk normality test. If a normal distribution was confirmed, a one-way repeated analysis of variance (ANOVA) with the Geisser–Greenhouse correction in case of violation of sphericity was used to determine whether g-level (1 g, Martian gravity and Lunar gravity) had any effects on joint kinematics and fascicle‒SEE outcomes (n = 8). If a significant effect of g-level was observed, Tukey’s post-hoc test was used to correct for multiple comparisons using statistical hypothesis testing (1 g vs. Martian gravity, 1 g vs. Lunar gravity, and Martian gravity vs. Lunar gravity). If the data were not normally distributed, as was the case for the time of peak SEE length, fascicle velocity at the time of peak SEE length, and stride length, the non-parametric Friedman test, and Dunn’s post test were used (n = 8). The statistical analysis was performed in GraphPad Prism (v 7.04) with α set to 0.05. Data are reported as mean (± standard deviation). Furthermore, effect sizes f(U) for the ANOVA were calculated using the G*Power software version 3.1.9.4^41^. Effect sizes for the respective post-hoc comparisons are presented as Cohen’s d. Thresholds of d = 0.2, d = 0. 5 and d = 0.8 were defined as small, moderate, and large effects^42^. While the data (mean ± standard deviation) acquired at 1 g have already been presented in a previous publication^19^, the differences to simulated Martian and Lunar gravity as well as between Mars and Moon have not been published elsewhere.

## Data Availability

The datasets generated and/or analyzed in the course of the current study are available from the corresponding author upon request.

## Abbreviations

g: Gravitational acceleration
GM: Gastrocnemius medialis
ISS: International Space Station
MTU: Muscle-tendon unit
PTS: Preferred walk-to-run transition speed
SEE: Series elastic element
VTF: Vertical treadmill facility
